# The Role of Nutrition in Cardiovascular Disease: Current Concepts and Trends

**DOI:** 10.3390/nu15051064

**Published:** 2023-02-21

**Authors:** Dimitra Tyrovola, Stergios Soulaidopoulos, Costas Tsioufis, George Lazaros

**Affiliations:** Cardiology Clinic, Hippokration General Hospital, School of Medicine, National and Kapodistrian University of Athens, 11527 Athens, Greece

Cardiovascular disease (CVD) is the leading cause of mortality worldwide [1]. The prevalence of CVD doubled from 1990 to 2019, and a steady increase in CVD deaths was observed, rising from 12.1 million in 1990 to 18.6 million in 2019 [2]. It is anticipated that CVD will remain the leading global cause of mortality, resulting in approximately 23 million deaths by 2030 [3]. 

Regarding prevention measures, it is estimated the adoption of healthy lifestyle choices reduces the risk of myocardial infarction (MI) by 81–94% [4,5,6], whereas treatment with pharmacotherapies alone results in a 20–30% reduction [7]. Accordingly, nutrition is the most important behavioral factor in preventing premature CVD death and disability, surpassing smoking abstinence and physical activity [8]. As a result, International Guidelines strongly recommend a healthy diet, with particular emphasis on the intake of fruits and vegetables, whole grains, fish, and legumes [9]. In contrast, intake of processed meats and fats should be minimized, aiming at efficacious control of CVD risk factors [9]. 

With respect to pathophysiology, it is well established that atherosclerosis and hypertension underly most of the cardiovascular disorders in the developed world, while both can be affected by dietary habits. There is also substantial experimental and clinical evidence indicating that atherosclerosis represents an inflammatory process characterized by a complex, dysfunctional interplay between the immune system and lipids [10]. Considered a crucial determinant of chronic systemic inflammation, obesity represents a major nutritional disorder which, owing to its dramatic global spread, is now considered an ongoing pandemic. Providing more support for the close link between dietary factors and inflammation, the adoption of plant-based diets has demonstrated significant effects on inflammatory biomarkers [11]. In this context, the Mediterranean (MED) diet (based mainly on whole grains, fruits, vegetables, beans, herbs, spices, nuts and healthy fats such as olive oil, moderate consumption of fish, poultry, egg and dairy and infrequent servings of red meats and sweets) as a healthy dietary pattern is well known for its anti-inflammatory potential.

The “ATTICA study”, which was conducted between 2001 and 2012 in Athens in Greece, revealed that the combination of MED with the adoption of a physically active lifestyle attenuates the inflammation process in healthy individuals, as indicated by a reduction in several inflammatory biomarkers [12]. From a clinical perspective, this was translated into a lower incidence of cardiovascular disease among individuals with greater adherence to MED [13]. 

Providing further support, Tyrovolas et al. recently reported an inverse association between a low anti-inflammatory diet and successful aging; in particular, the multi-adjusted analysis revealed that consuming a low anti-inflammatory diet was associated with lower levels of successful aging, irrespective of age, gender, residence area, smoking habit, and waist circumference [14]. In line with the latter observation, in 1995, Trichopoulou and colleagues disclosed a strong association between adherence to MED diet and overall survival. The main contributors to this association were moderate ethanol consumption, low consumption of meat products, high vegetable consumption, and high fruit and nut consumption [15]. Larger cohorts worldwide replicated the above-mentioned results, confirming the strong association between adherence to a MED diet and lower all-cause and cause specific (CAD, stroke, CVD, or cancer) mortality [16,17]. 

In a similar vein, in the Lyon Heart Study, during a mean follow-up of 46 months, all the composite outcomes, (namely cardiac death and non-fatal MI combined with additional major or minor secondary endpoints) were significantly reduced in the MED subgroup [18,19]. Among the potential benefits of the MED diet, an improvement in the left ventricular contractility and diastolic function was reported by Chrysohoou et al. [20,21]. 

Another well-known and popular proposed dietary patent depicting beneficial effects on the cardiovascular outcomes is DASH. This diet was initially developed as a dietary approach to lower blood pressure and is based on fruits, vegetables, and low-fat dairy foods. This dietary approach has been shown to decrease incident cardiovascular disease by 20%, coronary heart disease by 21%, stroke by 19%, and diabetes by 18% in prospective cohort studies. A favorable effect on blood pressure, lipid levels, blood glucose, and body weight has also been demonstrated in controlled trials [22]. Considering the remarkable effects of DASH diet on CVD, in 2013, the AHA Guidelines on lifestyle management to reduce cardiovascular risk recommended the DASH diet due to the “strong” level of evidence that it reduced CVD [23]. In a similar vein, the traditional Japanese dietary pattern (rich in fish, seaweed, soybean products, vegetables, and green tea), was shown to substantially reduce CVD mortality after adjustment for potential confounders in a seven-year prospective cohort study. Nevertheless, some components of the Japanese diet (i.e., salt) may account for an increase in the risk of hypertension [24]. 

To further address the role of nutrition habits on CVD outcomes, a 2012 meta-analysis by Huang et al., which included a total of 124,706 participants, showed significantly lower mortality from coronary artery disease (CAD) in vegetarians compared to non-vegetarians [25]. In the Physicians’ Health Study I (1982–2008)—a prospective cohort study of 20,900 men who were healthy at baseline—consumption of breakfast cereals, fruits, and vegetables was associated with a lower lifetime risk of heart failure [26]. 

The landmark Framingham Offspring Study, after 18 years of follow-up, demonstrated that each additional daily serving of ultra-processed foods was associated with a 7% increase in the risk of incident CVD [27]. Likewise, the French NutriNet-Santé cohort study showed that increased consumption of ultra-processed foods was associated with a 12% increased CVD risk [28]. Furthermore, there is convincing evidence from meta-analyses that specific ultra-processed products (processed meat, sugar-sweetened beverages) and nutrients that are abundant in ultra-processed foods (trans fats, sodium) enhance CVD risk [29]. Based on these findings, current dietary guidelines recommend saturated fat intakes below 10% of total energy intake for both people with diabetes and general population [30]. 

Excessive energy consumption and fat intake increases the risk of obesity and may, paradoxically, be combined with deficiency of essential micronutrients. In fact, contrary to previous findings, obesity seems to represent a new type of malnutrition, at least in the developed world. This may be the result of unhealthy eating habits along with malabsorption and altered metabolism of micronutrients following low-grade systemic inflammation promoted by obesity [31]. Poor dietary patterns linked to obesity may include limited access to nutrient-rich, high-quality foods which can be determined by socioeconomic factors. Indeed, diet quality level decreases with socioeconomic status, which is reflected in the higher prevalence of overweight and obesity [32]. Likewise, dietary and behavioral aberrations, along with insufficient access to modern therapeutic modalities, are considered to partially account for high cardiovascular disease prevalence among individuals of lower socioeconomic status. Without overlooking integrated management of classical risk factors, educational interventions aiming to improve eating behaviors are crucial to reducing cardiovascular disease [33]. 

Interestingly, some studies have investigated the association of nutrition with psychological parameters. In this context, Greco A et al. enrolled 275 consecutive patients affected by ACS, who received up to 5 years of follow-up. They studied the role of both psychological (anxiety and depressive symptoms) and environmental (seasonal variations) factors as predictors of the longitudinal trajectories of healthy behaviors in terms of diet and physical activity [34]. More anxious patients, who were more concerned about their health, maintained healthy behavior over time [34]. Recently, a healthy lifestyle, characterized by healthy dietary habits, was associated with a slower memory decline over a 10-year follow-up period in a Chinese cohort composed of 29,072 cognitively normal older individuals. Notably, the favorable effect of healthy lifestyle on cognitive function was not affected by the presence of genetic predisposition to memory decline, as assessed by the detection of the apolipoprotein E allele, which is considered to correlate with earlier and more progressive impairment of cognitive ability [35].

In conclusion, it is currently well established that a healthy diet is the cornerstone for CVD primary and secondary prevention. Optimum nutritional strategies promote longevity, reduce the risks of diabetes mellitus, arterial hypertension and stroke, prevent obesity, and thus reduce the risk of CVD. Nutrition should play a central role in prevention of CVD as a non-medicinal agent. Μore educational strategies should be implemented to emphasize the paramount importance of dietary habits in healthy life and aging. 
